# Use of prophylactic antibiotics following tube thoracostomy for blunt chest trauma in the prevention of empyema and pneumonia

**DOI:** 10.5249/jivr.v6i2.11

**Published:** 2014-07

**Authors:** Mohammad Bagher Heydari, Mohammad Ali Hessami, Khosro Setayeshi, Fatemeh Sajadifar

**Affiliations:** ^*a*^Department of Surgery, Imam Reza Hospital, Kermanshah University of Medical Sciences, Kermanshah, Iran.; ^*b*^Imam Hosain Hospital, Kermanshah, Iran.

Chest injury is a common problem in patients sustaining blunt or penetrating trauma.^1^ Thoracic wounds account for 20-25% of all trauma deaths. Only 10-15% of all chest wounds require tube thoracostomy, whereas the remaining 85% can be managed with a closed tube thoracostomy.^2^ A major morbidity associated with this therapeutic device is empyema. The role of prophylactic antibiotics in reducing the incidence of this complication is controversial. Multiple factors contribute to the development of posttraumatic empyema. These factors include the conditions under which the tube is inserted (emergent or urgent), the mechanism of injury, retained hemothorax and ventilator care.^3-8^

The primary goal of prophylactic antibiotic use in injured patients requiring tube thoracostomy is to reduce the incidence of empyema and its associated morbidity. The primary benefit must be significant because of the risk of the emergence of resistant organisms with excessive use of antimicrobials. In addition, cost is a major concern in the current health care market. The above-mentioned concerns were the reasons for performing this study.

**Patients and methods**

This study is a randomized controlled trial. It took place over a 2-year period from June 2005 to June 2007. Patients aged 8-72 years with traumatic hemopneumothorax following blunt chest trauma and receiving chest tube placement were enrolled for the trial. Patients were excluded if they had penetrating chest trauma, needed to receive different antibiotics because of other injuries or had known immune-compromising disorders. The patients were classified as group-A and group-B randomly, with 54 cases out of 104 being assigned to group-A. The remainder (50 cases) was assigned to group-B. Since there are currently no clear-cut recommendations regarding antibiotic use in patients requiring tube thoracostomy to treat chest injury, there was no ethical deviation in this study. Group-A received 2gr of Cefazolin for the first 24 hours and group-B received a placebo. Patients were then followed daily for signs of empyema or pneumonia. Patients then received a telephone follow-up at 3 months after discharge evaluating for delayed evidence of empyema or pneumonia. In this study empyema is defined as a positive pleural culture or purulence within the thoracic space in conjunction with elevated white blood cell count and /or fever. Also, pneumonia was defined as evolving infiltrate on chest radiograph 24 hours after inserting the chest tube with either purulent sputum or a positive sputum culture. 

Our sample consisted of 75% males and 25% females with an average age of 39.6 years. The indication for tube placement was pneumothorax in 74 (69.2%), hemothorax in 20 (19.2%) and hemopneumothorax in 12 (11.5%). Totally 112 chest tubes were inserted. Eight of these patients received bilateral tubes. The average duration of tube placement was 6.8 days. Six patients developed pneumonia, 2 in group-A, 4 in group-B (p = 0.3). One patient from group-B developed empyema (p=0.48). 

Overall, this study revealed that prophylactic antibiotics did not significantly reduce the incidence of empyema or pneumonia in patients with blunt chest trauma. The use of prophylactic antibiotics for the prevention of empyema and pneumonia after tube thoracostomy remains a controversial issue in the trauma literature. While a number of studies show favorable effects, several reports have shown no benefit. ^6-8^ In our study the incidence of empyema was very low. Having prescribed prophylactic antibiotics to a large numbers of patients, we managed to prevent just a single empyema. 

We concluded that prophylactic antibiotic administration did not significantly reduce the incidence of empyema or pneumonia in these patients. Therefore, considering the emergence of resistant organisms and the cost and benefit, it seems that prophylactic antibiotics should not be administered in the management of chest tubes for blunt chest trauma; however, larger and more comprehensive studies should be performed to confirm this. 
